# Assessing the impact of novel hybrid floating breakwater-WEC systems on hydrodynamic performance and sustainable energy outputs

**DOI:** 10.1038/s41598-026-37290-8

**Published:** 2026-02-18

**Authors:** Bayan Hamed, M. Elkiki, Sherif Abdellah, Yassen El.S. Yassen, Reda Diab

**Affiliations:** 1 Civil Engineering Department, Faculty of Engineering, Port Said University, Port Said, Egypt; 2 Mechanical Power Department, Faculty of Engineering, Port Said University, Port Said, Egypt; 3 Irrigation and Hydraulics Engineering Department, Faculty of Engineering, Mansoura University, Mansoura, Egypt; 4 Faculty of Engineering, Mansoura National University, Mansoura, Egypt; 5 Coastal/harbors Engineering, Civil Engineering Department, Faculty of Engineering, port Said University, Port said, Egypt

**Keywords:** Floating breakwater, Wave energy converter, Oscillating water column, Reflection coefficient, Transmission coefficient, Energy coefficient, Energy science and technology, Engineering, Physics

## Abstract

Developing novel low-reflection structures, such as Oscillating Water Column (OWC) wave absorbers, provides a promising solution for enhancing harbor berthing safety. OWCs present the dual advantage of reducing wave reflections while simultaneously capturing wave energy. This study experimentally investigates the reflection characteristics, efficiency of wave energy extraction, and power dissipation behavior of OWC absorbers with different rear wall configurations. Furthermore, it investigates variations in rear wall geometry, incident wave height, and the well turbine located inside the air chamber, which converts wave power into pneumatic power. Controlled wave flume experiments at the University of Port Said were conducted on four models. Key performance parameters analyzed include the dissipation coefficient (C_d_), energy coefficient (C_e_), transmission coefficient (C_t_), reflection coefficient (C_r_), and pressure coefficient (C_p_). The effects of different draughts, water depths, and air pressure fluctuations inside the pneumatic chambers were also examined. Results indicate that rear wall geometry significantly affects reflection and dissipation rates. Model-D achieved optimal performance at a water depth of 0.30 m with a front wall draught (d_1_) of 0.10 m, exhibiting low reflection (C_r_ = 0.139), high energy dissipation (C_d_ = 0.9), and a high wave energy conversion (C_e_ = 0.75; C_p_ = 0.85), making Model-D suitable for floating barriers in deep-water environments. Its superior wave energy dissipation enables effective operation under larger drafts and higher sea states.

## Introduction

Wave energy is a rich and cost-effective resource with significant potential to enhance the global renewable energy mixture. Various Wave Energy Converters (WECs) have been developed to capture this energy by converting the mechanical motion of waves into electrical power. These devices are classified according to their geometry, mode of operation, and location relative to the shoreline, as onshore, nearshore, or offshore systems. In terms of working principles, WECs are divided into point absorbers, oscillating bodies, overtopping devices, or Oscillating Water Columns (OWCs). Such classifications determine their energy capture efficiency and suitability for different marine environments^1–3^. Among these devices, OWC has been recognized as one of the most practical and well-studied types of WEC due to its simple configuration and minimal number of moving parts. An OWC consists of a fixed or floating hollow chamber that traps a column of air above a water surface open to the sea below. The vertical motion of waves causes the trapped air to compress and decompress, forcing airflow through an air turbine connected to an electrical generator^4^. The OWC’s simplicity, strong structure, and low maintenance requirements make it particularly suitable for integration into coastal or breakwater structures. Breakwater Wave Energy Converters (BW-WECs) are associated with the protective function of floating breakwaters, also presenting energy generation capability. Such hybrid systems effectively utilize the motion of floating bodies to dissipate wave energy while generating electricity. The Water Column (OWC)-based BW-WECs are considered highly promising for coastal protection and renewable energy harvesting due to their dual functionality and economic feasibility.sxds

Recent research has focused on the development and optimization of hybrid floating energy systems. For example^5^, proposed a V-shaped floating platform that integrates a wave energy converter (WEC) with a wind turbine to optimize both wind and wave energy utilization. Many tools, such as FAST ^6^, FORTRAN^6^, and ANSYS-AQWA^7^, were used to develop an integrated numerical analysis structure for investigating the dynamic behavior of the system. The results showed that the aerodynamic load reduces the platform’s wave speed and generates a coupling effect between external and internal floats, supporting the development of hybrid floating energy systems in practical applications. Similarly^8^, discussed the hydrodynamic interactions and design challenges associated with such floating energy systems.

Furthermore, several studies investigated combined porous and oscillating water column designs to improve hydrodynamic performance. For example^9^, demonstrated that integrating a porous plate with an OWC significantly reduced horizontal wave forces (up to 52%) while maintaining high conversion efficiency. Similarly^10^, analyzed the influence of three-dimensional effects, wave incidence angles, and submergence depth on energy efficiency, finding that 3D configurations enhance performance near the resonance frequency. Additionally^11^, used numerical simulations (Open FOAM) to study an OWC positioned above a horizontal plate, and utilized numerical modelling (Open FOAM) to investigate an OWC above the horizontal plate, showing that the inclusion of an immersed plate greatly improves energy capture and stability while achieving a reflection coefficient ranging from 0.8 to 0.45.

For air turbines, floating breakwaters are preferred due to their reasonable construction costs, low environmental impact, and flexibility^12^. The well’s turbine has simplicity and high generator compatibility, but it suffers from a limited operating range and high axial thrust^13^. ^14^ Introduced self-pitch-controlled guide vanes to reduce shock loss, though this increased the mechanical complexity to address this.

Over the years, various BW–WEC configurations have been investigated, based on structural elements, material selection, and hydrodynamic efficiency. For example^15^, proposed a Pentagonal Backward Bent Duct Buoy (PBBDB) with an OWC using two turbine generators, achieving an efficiency of up to 33.4% in regular waves. ^16,17^ explored box-type and wedge-shaped floating breakwaters, respectively, while^18^ showed that adding rotary pneumatic chambers significantly reduces wave transmission (C_t_ from 0.96 to 0.15) and enhances energy dissipation (C_d_ up to 0.51). Such results demonstrate the potential of integrating OWC-based systems within floating breakwaters for combined coastal protection and renewable energy generation.

Most of the previously OWC-WEC systems have focused on experimental studies to validate numerical models or demonstrating the actual hydrodynamic behavior of the system. However, most of these experimental data belong to large-scale or fixed OWC systems, while few studies integrate small-scale or floating OWC systems with breakwater structures. This study aims to experimentally evaluate hydrodynamic performance, energy conversion, and the design of floating breakwaters. This includes studying the impact of rear wall geometries on dynamic performance and air pressure fluctuations inside vertical chambers, as well as evaluating the efficiency of pneumatic systems and turbines.

This study introduces a new hybrid design for a floating breakwater that aims to enhance wave attenuation and energy generation, which has either been minimally or not previously assessed simultaneously. The final configuration represents a hybrid and innovative design optimized for both wave attenuation and power generation. It focuses on the effect of rear wall geometry, incident wave height, and front wall draft on the performance of OWC absorber for the water depth scenarios. The study investigates how the rear wall geometry affects the water oscillation in the chamber, pressure variations, anti-reflection capability, energy extraction efficiency, as well as dissipation of wave energy of the OWC-type absorber. The remainder of the paper is organized as follows: Section "Methodology", Experimental setup, data acquisition, and wave conditions. Section "Results and discussion", Results and discussions regarding the hydrodynamic performance of the BW-WEC, including transmission, reflection, energy dissipation, and pressure coefficient. Finally, section “Conclusions” xsprovides a summary of the main conclusions based on this study.

## Methodology

### Experimental configuration and data acquisition

**Fig. 1:**
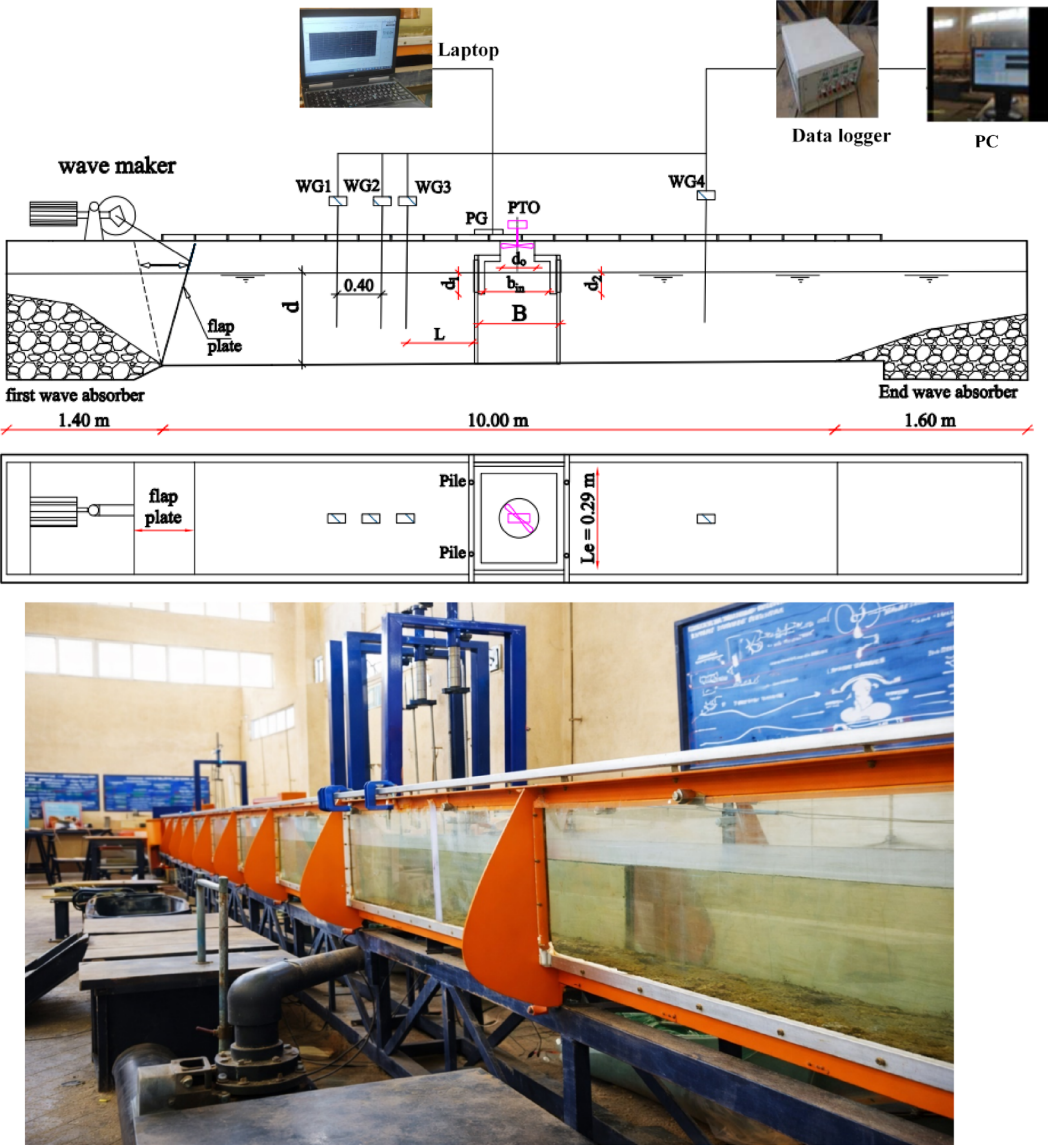
Experimental set up of the FB: Side view (top), plan view (middle), and photo (bottom).

**Fig. 2:**
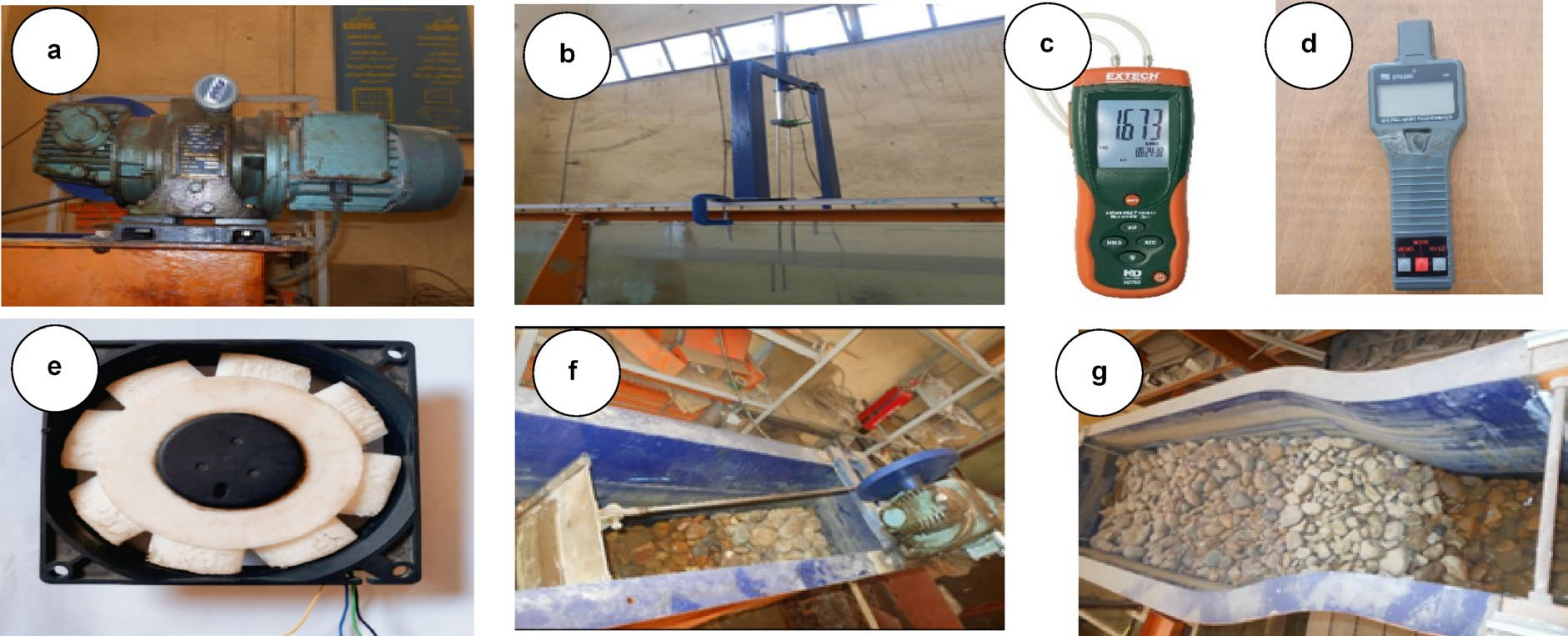
Instrumentation setup onboard the FBW-WEC models: (**a**) Wave maker, (**b**) Wave probe, (**c**) Pressure gauge, (**d**) Tachometer, (**e**) PTO and voltmeters, (**f**) First absorber, and (**g**) End absorber.

The experiments were carried out in the Wave Flume of the Fluid Laboratory, Faculty of Engineering, Port Said University (Egypt). The wave flume is 13 m long, 0.3 m wide, and 0.5 m high, as shown in Fig. 1. A flap-type wave maker was installed at one end of the flume to generate regular waves with 0.6 to 1 s periods and 4 to 12 cm high, respectively. Wave-absorbing beaches were located at both ends of the flume to reduce the wave reflection, as shown in Fig. 2. The Wave-absorbing beaches are made up of well-graded gravels having a gradual slope of 1:10.

### Floating breakwater experimental model

**Fig. 3:**
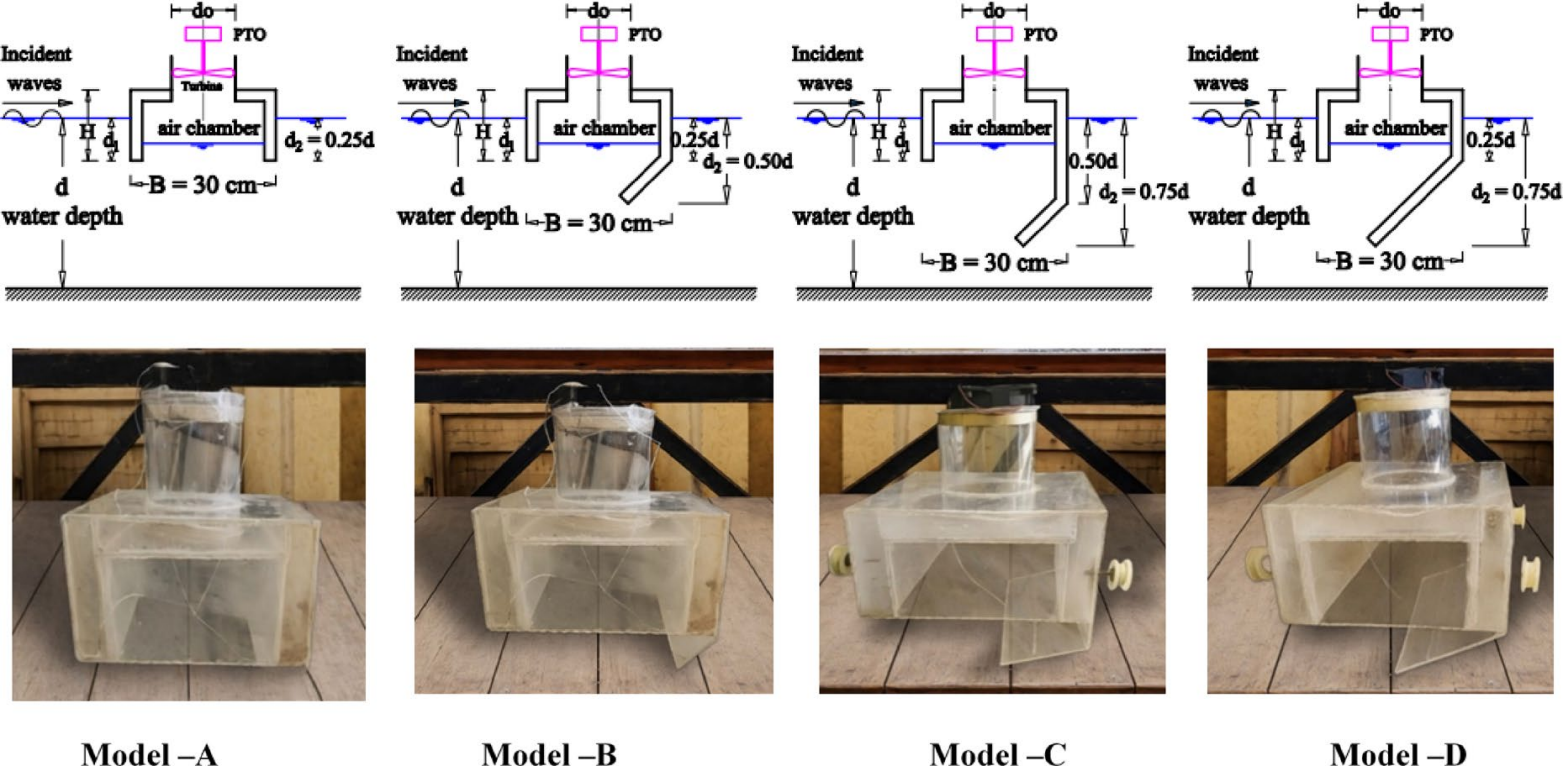
Tested breakwater models: (**a**) schematic details (top), and (**b**) Photo (bottom).

The suspended floating breakwater model developed in this study is a considerable departure from conventional symmetric designs. These FB-WECs utilize a modified (OWC) system consisting of three key enhancements: (1) a geometrically optimized rear wall, (2) a top-mounted airflow orifice, and (3) an integrated well turbine driven by oscillating pneumatic pressure. The FB-WEC was positioned at the center section of the flume, at 5 m from the wave makers. The main structures of the FB-WEC device models were constructed using flexible Perspex sheets with a thickness of 3 mm, which allowed the vibration of the water column to be visible. These structures were then fastened to four rounded aluminum piles to ensure stability. In this context, B represents the width of the OWC chamber, d_1_ is the front wall draft, and d_2_ is the rear wall draft, as detailed in Fig. 3. Therefore, the structure has a length of 0.29 m perpendicular to wave direction, 0.16 m height, 0.25 m internal OWC width, 0.3 m outer width, the diameter of the air cylinder is 0.11 m, and 0.11 m height. Four distinct configurations were evaluated: (a) Model-A simple pontoon, (b) Model-B short slope, (c) Model-C vertical rear wall followed by short slope, and (d) Model-D long slope. Experimental test status and wave properties in this study are presented in Table 1. In addition, the Reynolds number (Re) at the inlet of the FB-WEC models was calculated to determine the flow regime. The results show that Re ≈ 1.69 × 10^5^ to 1.03 × 10^6^, confirming that the flow around the floating breakwaters is fully turbulent. This information is essential for interpreting wave-structure interaction and energy dissipation.

**Table 1:** Experimental test conditions and wave characteristics.

Parameter	Tested condition
B	0.30m
L	0.29m
d_1_	0.1–0.08–0.06–0.04cm
d_2_	0.25–0.75d
d_1_/d_2_	1–0.3
d	0.3–0.25–0.20cm
Hi	0.116–0.032cm
Li	1.529–0.32cm
B/L	0.902–0.19
H/L	0.1–0.075
d/L	1.09 – 0.15
bin	0.25

Due to the limitations of the experimental setup, the model was scaled down to fit within the wave flume. To ensure that the test conditions accurately represent a real sea environment, selecting an appropriate scale is essential to minimize the impact of wave reflections from the flume walls. The model must achieve three fundamental types of similarity with the full-scale prototype: geometric, kinematic, and dynamic. The wave parameters used in the tank should also conform to these scaling principles. In this scenario, where inertial forces dominate over viscous forces, the Froude number takes precedence over the Reynolds number. The experimental setup was designed based on Froude’s similarity law to ensure dynamic similarity between the model and the prototype^19^. The scaling factors for length, velocity, and time were defined, and other quantities, such as acceleration, area, wave height, and wave length, were scaled as shown in Table 2. Preserving a constant Froude number, as given in Eq. (1) across the scaling process, is critical to maintaining kinematic and dynamic similarity^20^.

1 $$\text{Froude number }(\mathrm{Fr}) = \frac{{\mathrm{u}}_{\mathrm{p}}}{\sqrt{{\mathrm{gxL}}_{\mathrm{p}}}} = \frac{{\mathrm{u}}_{\mathrm{m}}}{\sqrt{{\mathrm{gxL}}_{\mathrm{m}}}}$$

where U_p_ is the prototype velocity, U_m_ is the model velocity, g is gravitational acceleration, L_p_ is the prototype wavelength, and L_m_ is the model wavelength.

**Table 2:** Summary of Froude scaling for the model and prototype.

Quantity	Prototype	Scale relationship	Scale factor
linear	L_p_/L_m_	S	20
Dimension			
Linear velocity	$v_{p} /v_{m}$	S^^(1/2)^	4.47
Acceleration	a_p_/a_m_	1	1
area	A_p_/A_m_	S^^2^	400
Wave height	H_p/_H_m_	S	20
Wavelength	$\left( {\lambda_{p} /\lambda_{m} } \right)$	S	20
Wave period	T_p_/T_m_	S^^(1/2)^	4.47

### Data acquisition system and data analysis

Preparations were made in the laboratory to ensure an accurate test of the model before each experiment. These preparations included validating the water depth, selecting an appropriate test duration, and calibrating the wave gauge. Each experimental setup was tested with a minimum of three runs under the same wave conditions to ensure reliability and repeatability. The data presented for all coefficients (i.e., transmission, reflection, etc.) are the average values from the replicated trials. Four HR Wallingford wave gauges were strategically positioned around the tested model to measure wave elevations. The wave probes consist of two thin, 0.1 mm wide, parallel electrodes made of stainless steel.

Three gauges (WG1, WG2, and WG3) were positioned on the incident side to measure the incoming and reflected waves, with distances of 0.4 m between WG1 and WG2**,** and 0.25 m between WG2 and WG3**.** The nearest gauge (WG3) was located 0.32 m in front of the breakwater model**.** A wave** g**auge (WG4) was positioned 2.0 m downstream of the model (wave absorber side) to measure transmitted wave heights (Ht). The wave reflection coefficient (C_r_​) was determined using the two-point method described by^21^. Although four wave gauges were installed along the flume, the use of a single gauge was considered sufficient, as the wave field in this region is predominantly progressive with negligible reflection, consistent with the findings of^22^**.** According to^23^, when the wavelength is relatively long compared to the chamber’s horizontal dimensions, the surface motion at one point can effectively represent the entire surface variation inside the chamber. Therefore, the use of a single gauge downstream of the OWC was considered sufficient to characterize wave transmission without significant influence from beach reflections. The wave data were recorded at approximately 34 Hz and for 20 s per run to ensure accurate and reliable measurements. A Pressure Gauge (PG) was installed on top of the pneumatic chamber to monitor air pressure changes, and it was connected to a laptop for data extraction.

**Fig. 4:**
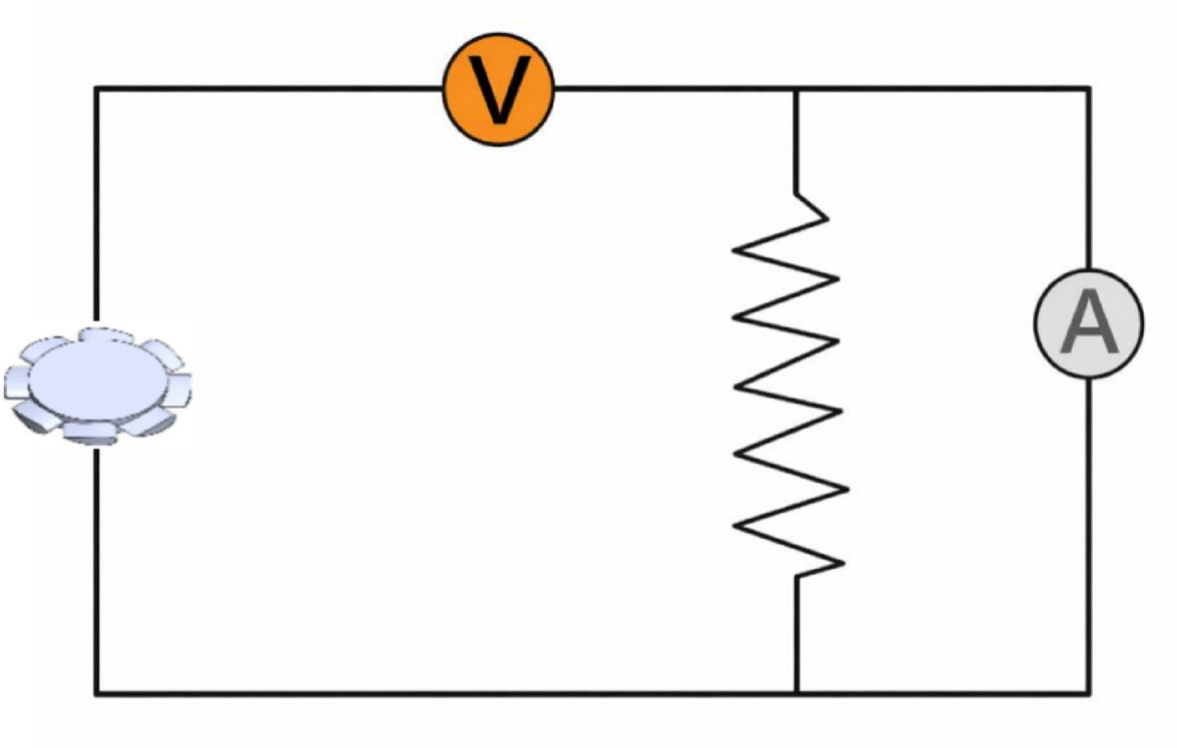
The electrical circuit for the well turbine.

The developed circuit is designed to acquire real-time data to measure the voltage and current generated by the turbine during operation, thereby enabling the calculation of electrical power consumption, as shown in Fig. 4. To determine the generator’s mechanical power (P), two instruments were employed: a voltmeter to measure the voltage (v) across the load, and an ammeter to measure the current (i) flowing through the circuit to determine the generator’s mechanical power. The voltmeter was connected in parallel with the turbine, while the ammeter was connected in series. The mechanical power output (P) was calculated as the product of the measured current (i) and voltage (v), expressed as:

2 $$P=i \text{x v}$$

**Fig. 5:**
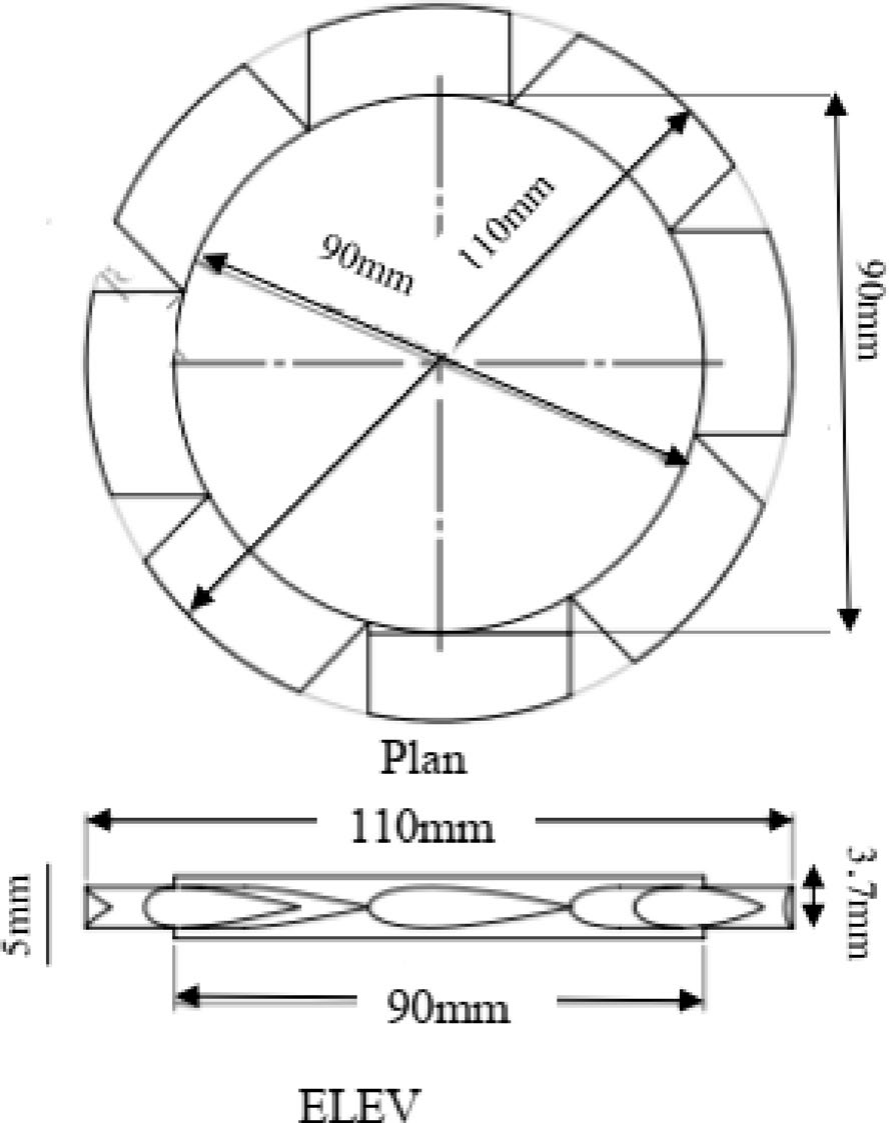
Schematic of the plan, ELEV, and 3D well turbine.

**Fig. 6:**
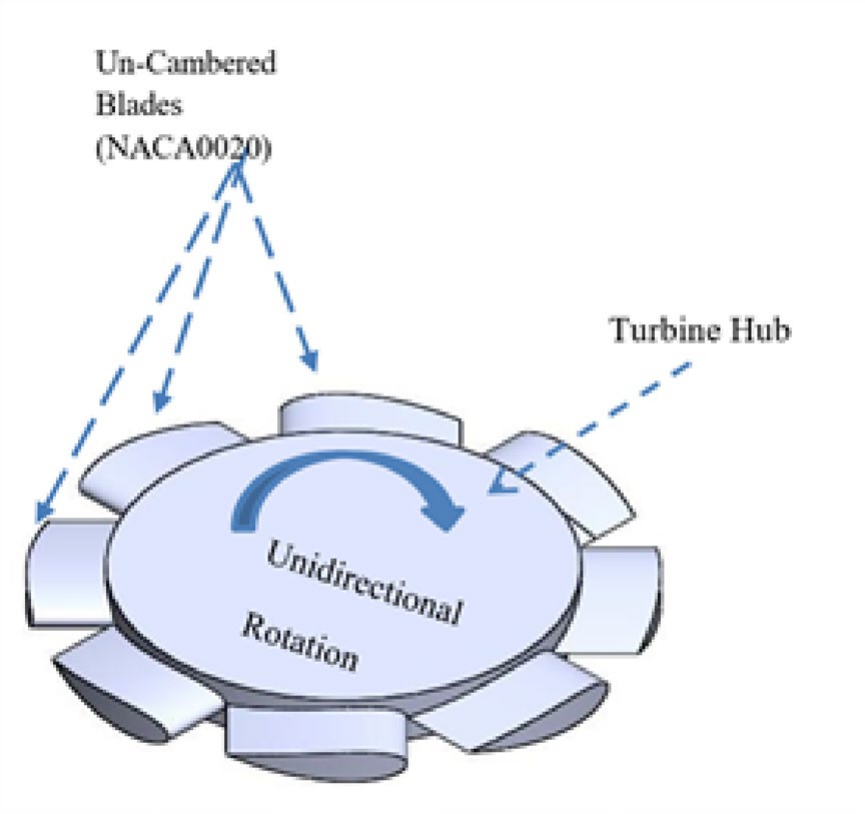
Well turbine 3D Schematic description and main characteristics.

Table 3 shows the standard well turbine specifications used in this study. The turbine includes eight NACA 0020-profile blades, with a hub radius of 45 mm and a blade chord of 25 mm. The tip clearance is 2 mm, and the blade span is 10 mm, as illustrated in Figs. 5 and 6. This turbine, as a simplified PTO model, does not replicate a specific turbine type but is designed to produce a consistent, measurable damping effect. The turbine is effective in oscillating flow because it has guiding vanes on both sides of the rotor.

**Table 3:** The standard turbine specifications.

Blade profile	NACA0020
Number of blades, z	8
Blade chord, C	25mm
Maximum blade thickness, t	3.75mm (18% C)
Blade hub radius, Rh	45 mm
Blade tip radius, Rt	55mm
Tip clearance	2mm
Turbine blade span, S	20 mm
Stagger angle, β	90°

#### Dimensional analysis

Key parameters characterizing the hydrodynamic performance of the breakwater include the incident-wave height (Hi), reflected-wave height (Hr), transmitted-wave height (Ht), and the internal chamber power amplitude (E). The functional relations defining the breakwater’s performance are formally given by Eq. (3):

3 $$\mathcal{F}\, = \, (Hi,Hr, \, Ht,E, \, B,d,d_{1} , \, d_{2} , \, L, \, g,\gamma_{w} , \, d_{0} , \, A_{0} , \, V)$$

Key parameters include the model width B, water depth d, front wall draft d_1_, rear wall draft d_2_, wavelength L, gravitational acceleration g, the specific weight of water γ_w,_ the diameter of pneumatic air d_0_, turbine swept area A_0_, and flow velocity V. The following dimensionless relationships are derived through π-theorem dimensional analysis by Eq. (4):

4 $$C_{r} , \, C_{t} , \, C_{e} , \, C_{p} = \, \mathcal{F} \, \left( {d_{1} /d, \, d_{2} /d, \, B/L, \, Hi/L} \right)$$

Equation (5, 6, 7, 8, and 9) show hydrodynamics coefficients^24^,^25^ include the reflection coefficient C_r_, the transmission coefficient C_t_, the energy coefficient C_e_, the pressure coefficient C_p_, and the dissipation coefficient C_d_. These coefficients are dimensionless parameters derived from fluid mechanics principles and have been widely adopted in wave–structure interaction studies^26^, defined as:

5 $$\text{Cr }=\frac{ Hr}{Hi}$$

6 $$\text{Ct }= \frac{Ht}{Hi}$$

7 $$\mathrm{Ce}=\frac{P}{0.5 \rho AV3}$$

8 $$\text{Cp }=\frac{\Delta p}{0.5\rho gHi}$$

9 $$C_{d}^{{}} = \, 1 - \, C_{r}^{2} {-} \, C_{t}^{2} - \, C_{e}$$

where ρ is water density, A = πr^2^ is the turbine swept area, P is the mechanical power, and V is the flow velocity, where V = $\frac{L}{T}$.

#### Acquisition of data and error analysis

An experimental reading was taken from various measuring devices at the intake and outlet of the OWC. The pressure gauge, voltmeter, ammeter, and wave gauge were used to measure the pressure inside OWC, power, and wave height, respectively.

Table 4 lists the measuring tools and their related accuracy. The standard uncertainty, U*,* for every measuring tool is expressed using the following formula from^27^:

**Table 4:** Accuracy, variables, and uncertainty of the employed tools.

Tools	Variables	Accuracy	Ranging	Standard uncertainty
HR Wallingford wave gauges	Height	± 0.001 m	-0.5 to + 0.5 m	0.00058 m
Pressure gauge	Pressure	± 0.05 bar	up to 50 bars	0.03 bar
Tachometer: Teston DT5350 Digital Hand Tachometer	Shaft rotational speed	± 1.0 rpm	20 rpm to 5500 rpm	0.58 rpm
Digital volt-ampere meter	Volt and ampere	± 0.01V and ± 0.01 A	DC 0 ~ 100 V and 0 ~ 20 A	5.77 × 10^–3^ V and A

10 $$\mathrm{U}=a{/3}^{1/2}$$

Accuracy is denoted by *a* in this case^27^**.**

Evaluation uncertainty is characterized as anxiety about the validity of measured values^28^**.** A function *Φ* with ‘*n*’ independent linear parameters, like Φ = f (λ_1_, λ_2_, ….…, λ_n_), can have its uncertainty calculated from^29^ .

11 $$\partial\Phi =\sqrt{{\left(\frac{\partial\Phi }{\partial {\uplambda }_{1}}\partial {\uplambda }_{1}\right)}^{2}+{\left(\frac{\partial\Phi }{\partial {\uplambda }_{2}}\partial {\uplambda }_{2}\right)}^{2}+\dots +{\left(\frac{\partial\Phi }{\partial {\uplambda }_{\mathrm{n}}}\partial {\uplambda }_{\mathrm{n}}\right)}^{2}}$$

For instance, Eq. (11) is used to determine the maximal uncertainty for any system efficiency,*η*, as indicated below:

12 $$\upeta =\mathrm{f}\left(\dot{\mathrm{W}},\text{ Q}\right)=\mathrm{f}\left(\mathrm{p},\mathrm{T},\mathrm{u}\right) \frac{\partial\upeta }{\upeta }=\pm \sqrt{{\left(\frac{\partial \mathrm{p}}{\mathrm{p}}\right)}^{2}+{\left(\frac{\partial \mathrm{T}}{\mathrm{T}}\right)}^{2}+{\left(\frac{\partial \mathrm{u}}{\mathrm{u}}\right)}^{2}}=\pm 0.012$$

Based on Eq. (12), the calculated uncertainty of η is 0.012%, suggesting that the experimental data are reliable. Similarly, the uncertainties of power and wave gauge are ± 0.012% and 0.0032%, respectively.

## Results and discussion

This section presents an empirical investigation of the capture width ratio of (BW-WEC) under various accident waves and applied PTO damping levels. For consistent comparison, Model-A geometry was tested with a fixed length between the front wall and rear wall, which will serve as the case study. This section compares the experimental results of Models-A, B, C, and D to examine how different rear wall shapes affect hydrodynamic characteristics such as transmission, reflection, energy dissipation, and pressure coefficient. All results derive from experimental investigations, with the primary objective of establishing design principles for high-efficiency hybrid BW-WEC systems.The effect of turbine rotational speed on voltage, power, and electrical torque

**Fig. 7:**
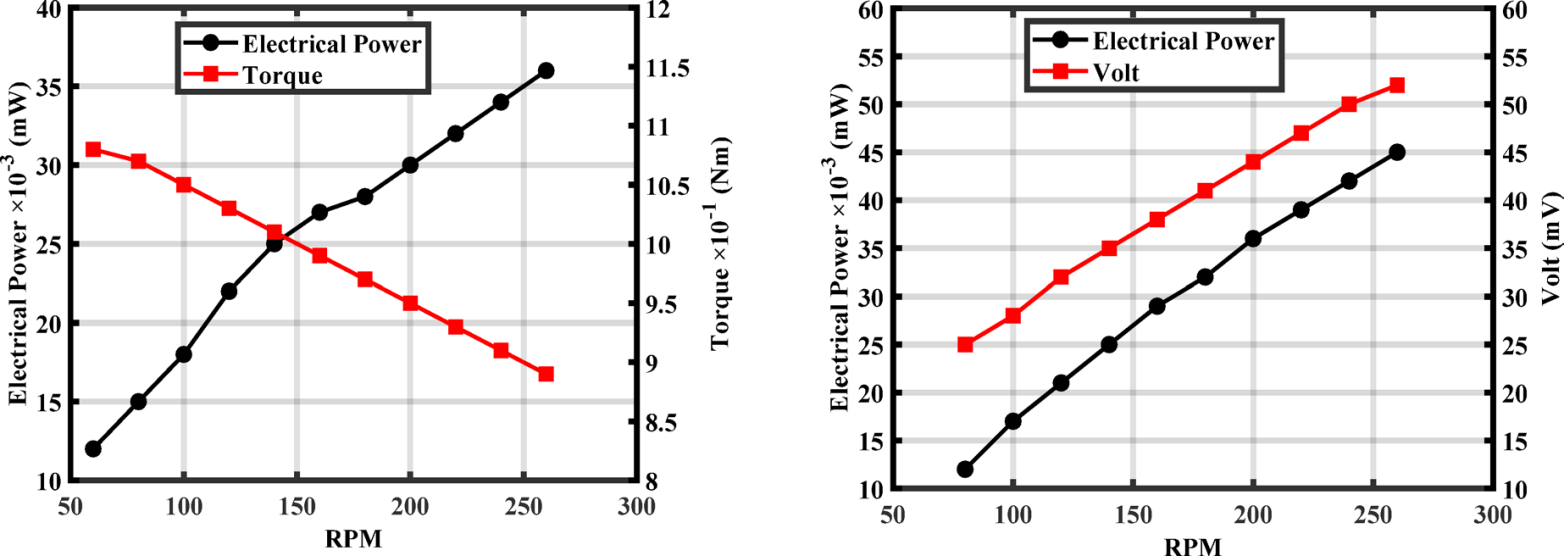
Variation of electrical power, torque, and voltage with rotational speed (RPM).

Figure 7 illustrates the relationship between the turbine’s rotational speed (RPM) and the values of voltage, electrical power, and torque. It is observed that the voltage exhibits a proportional relationship with increasing speed, due to the electromotive force induced being directly proportional to the rate of magnetic field cutting according to Faraday’s law. In contrast, the electrical power demonstrates non-linear behavior, approximately proportional to the square of the speed, as power is the product of voltage and current, both of which increase with speed. Meanwhile, the torque increases linearly with speed, derived from the fundamental relationship between power, torque, and angular velocity (P = T × ω), indicating a gradual increase in the mechanical load on the shaft. These results highlight the critical importance of controlling rotational speed through governor systems to ensure the stability of generated voltage and power, and to maintain the turbine within a safe and efficient operational range.

### Hydrodynamic performance of the BW-WEC

#### Wave energy spectrum

**Fig. 8:**
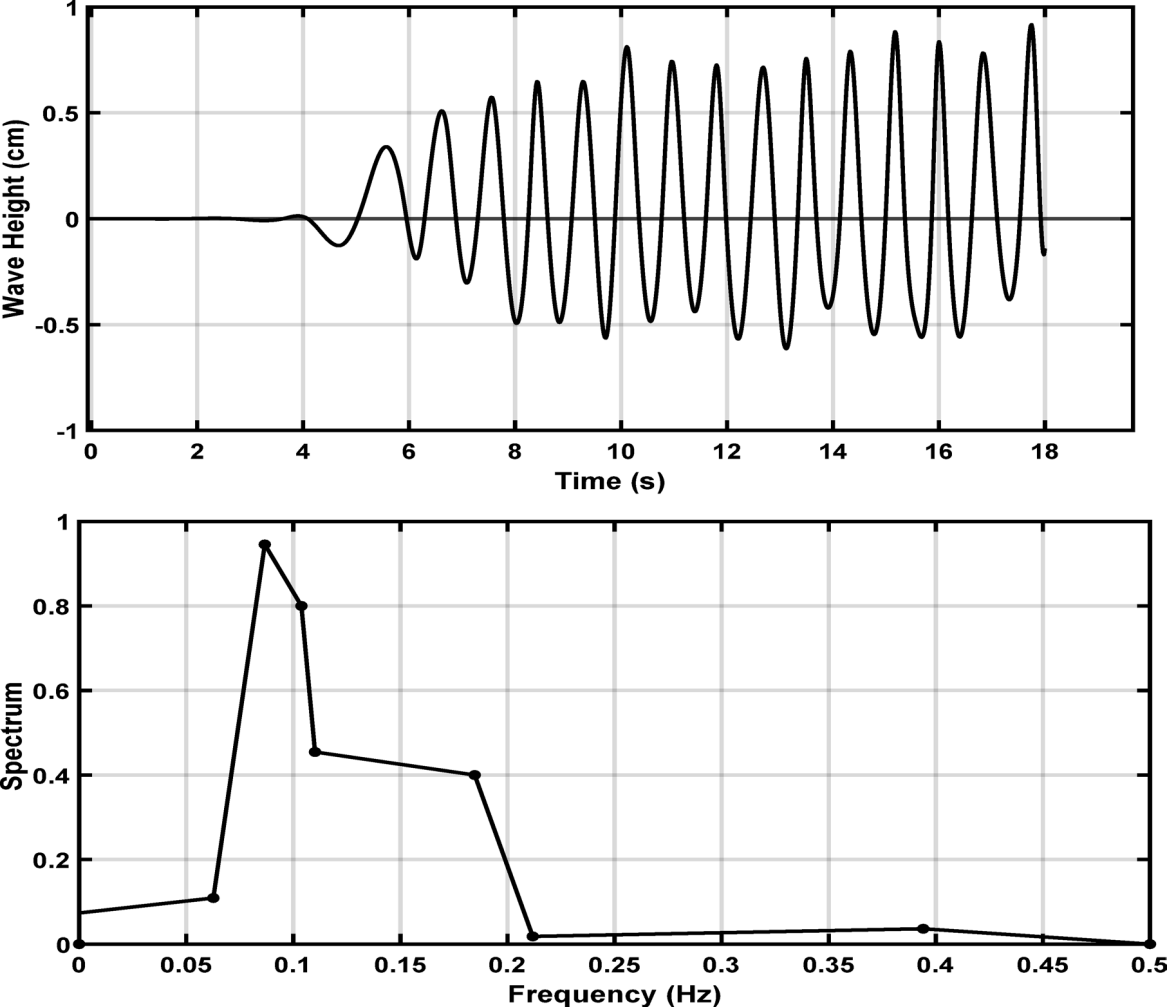
Typical time series of water level variation for a standing wave, obtained from flume measurements using a wave gauge, and the corresponding wave energy spectrum in the frequency domain.

**Fig. 9:**
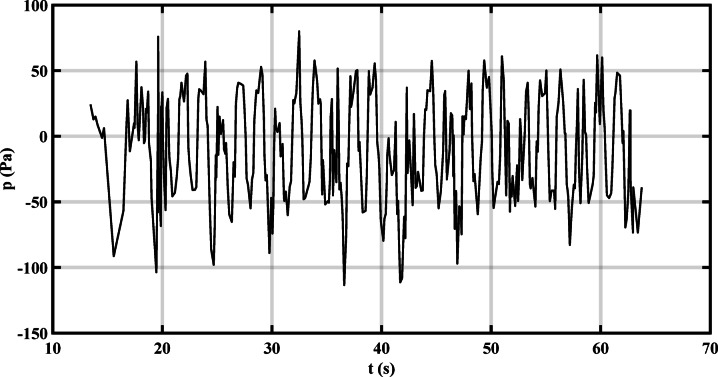
Pressure drop time series corresponding to a regular wave test.

Time series for the variation of water levels and pressure drop for the standing wave obtained from different breakwaters and Wave energy spectrum in the frequency domain (Hi = 11.6 cm, Li = 144.7 cm, and T = 1.52 s at H = 30 cm) are presented in Figs. 8 and 9. Even when regular waves are generated, the measured energy spectrum does not exhibit a perfectly sharp peak due to experimental imperfections, reflections from tank boundaries, measurement noise, and the finite duration of the signal. These factors spread the energy slightly around the main frequency, resulting in a broader spectral peak instead of a singular sharp spike^30–32^.

#### Wave reflection coefficient

**Fig. 10:**
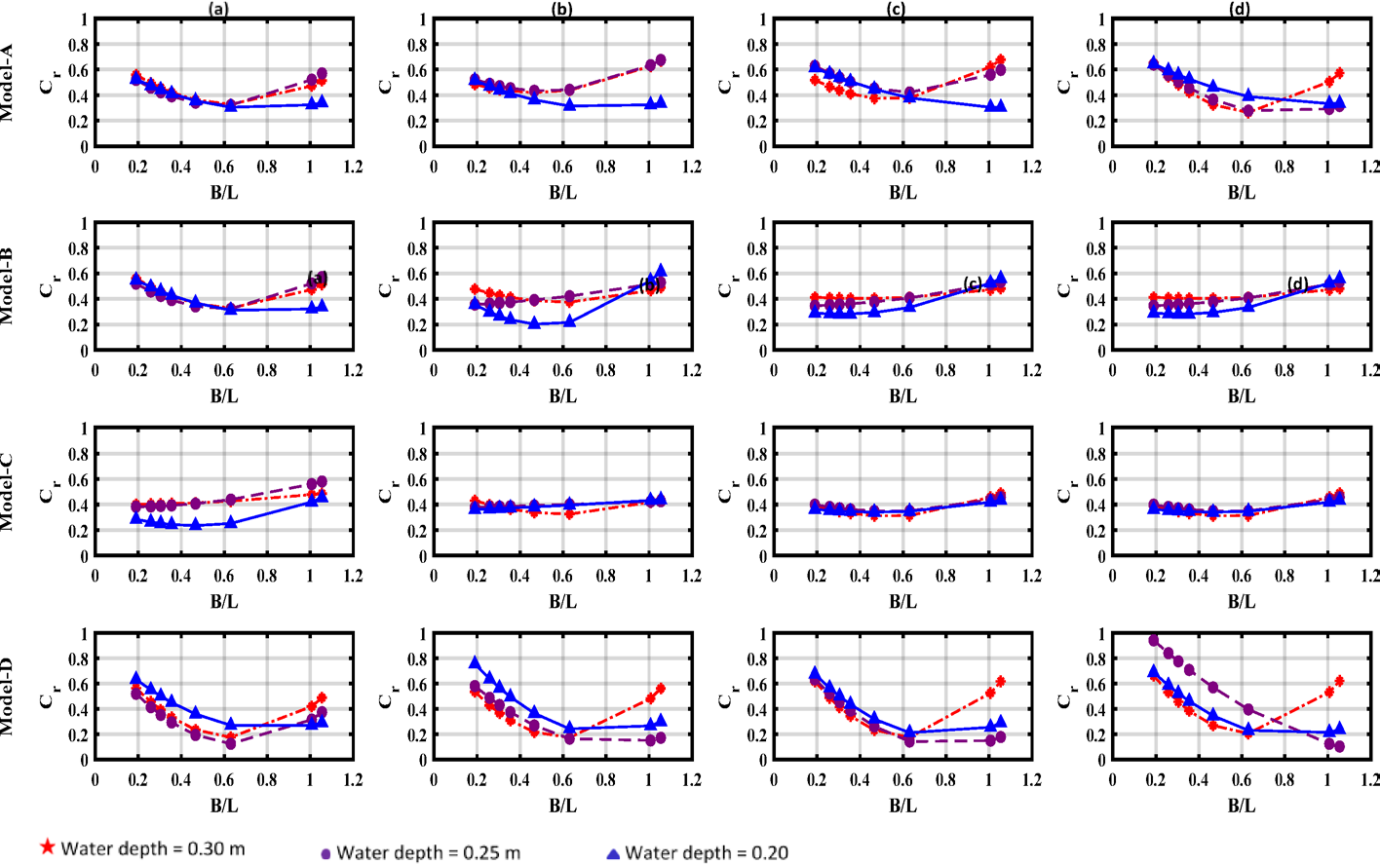
Variation of the reflection coefficient (Cr) with the relative width (B/L) across tested water depths; (**a**) draught = 0.04 m, (**b**) draught = 0.06 m, (**c**) draught = 0.08 m, (**d**) draught = 0.10 m.s

Figure 10 illustrates the variation of (C_r_) with B/L across different water depths. A consistent trend is observed: (C_r_) initially decreases with increasing B/L, reaching a minimum between B/L = 0.42 and 0.62 before rising again^33^**.**

For larger draft depths (d_1_ = 0.10 m), (C_r_) remains relatively high despite the increase in B/L. This behavior indicates reduced wave energy dissipation and thus high reflections. In contrast, intermediate drafts (d_1_ = 0.06 m and d_1_ = 0.08 m) exhibit a more significant reduction in (C_r_) as B/L increases, due to greater space for wave energy dissipation below the water surface, leading to lower reflection values. In the case of shallow drafts (d_1_ = 0.04 m), the lowest (C_r_) values were recorded at higher B/L ratios, as a smaller draft depth facilitates greater wave energy absorption, thereby significantly reducing reflected energy. These results confirm that deeper drafts contribute to more effective wave dissipation and improved breakwater performance.

Most of the experimental tests were conducted in intermediate water depths (1/20 < d/L < 1/2), with three deep-water conditions (d/L > 1/2). At intermediate depths, the reduced L for a given wave period^34^, thus producing downward-convex Cr curves, indicating that (C_r_) was higher in very shallow and deep water, the lowest (C_r_) values occur at intermediate depths.

The observed decrease in (C_r_) at higher B/L ratios can be attributed to two main factors (i) reduced wave reflection due to overtopping phenomena, and (ii) increased energy dissipation due to enhanced wave-structure interactions in scaled models. Among the tested models, Model-C and D showed promising performance in enhancing the BW–WEC efficiency. This can be attributed to the rear wall geometry, which enhances wave interaction and improves water column motion inside the pneumatic chamber. Additionally, the inclined rear wall helps to reduce the impact of reflected waves, thereby decreasing mechanical pressure and enhancing the breakwater’s resistance to harsh environmental conditions.

The remaining wave energy is distributed among several processes (i) viscous and turbulent dissipation, (ii) power extraction at the PTO, (iii) potential energy storage via chamber free-surface heave, (iv) rear-wall reflection, (v) transmission beyond the rear wall^21^. Although the curve increases quickly after reaching the minimum value, a relatively broad low reflection frequency band is observed due to a relatively small minimum (C_r_ = 0.138 for d = 0.25 m, d_1_/d = 0.27). Additionally, the hyperbolic cosine determines the particle velocity distribution along the vertical direction.

For short waves with longer wavelength, the energy concentration near the free surface reduces the hydrodynamic significance of structural draft depths on reflection coefficients. Conversely, for longer wavelengths, the energy-carrying fluid layer becomes thicker, which modifies the interaction between the wave and the rear wall. Thus, in Model-A and Model-B, the rear wall is partially immersed; the straight portion can redirect some of the waves away, while the small sloped portion dissipates the rest. Since wave reflection in Model-A and Model-B showed little sensitivity to changes in the wave period, the energy loss was mainly due to friction and turbulent flow separation, which were concentrated near the sharp edges of the breakwater.

#### Wave transmission coefficient

**Fig. 11:**
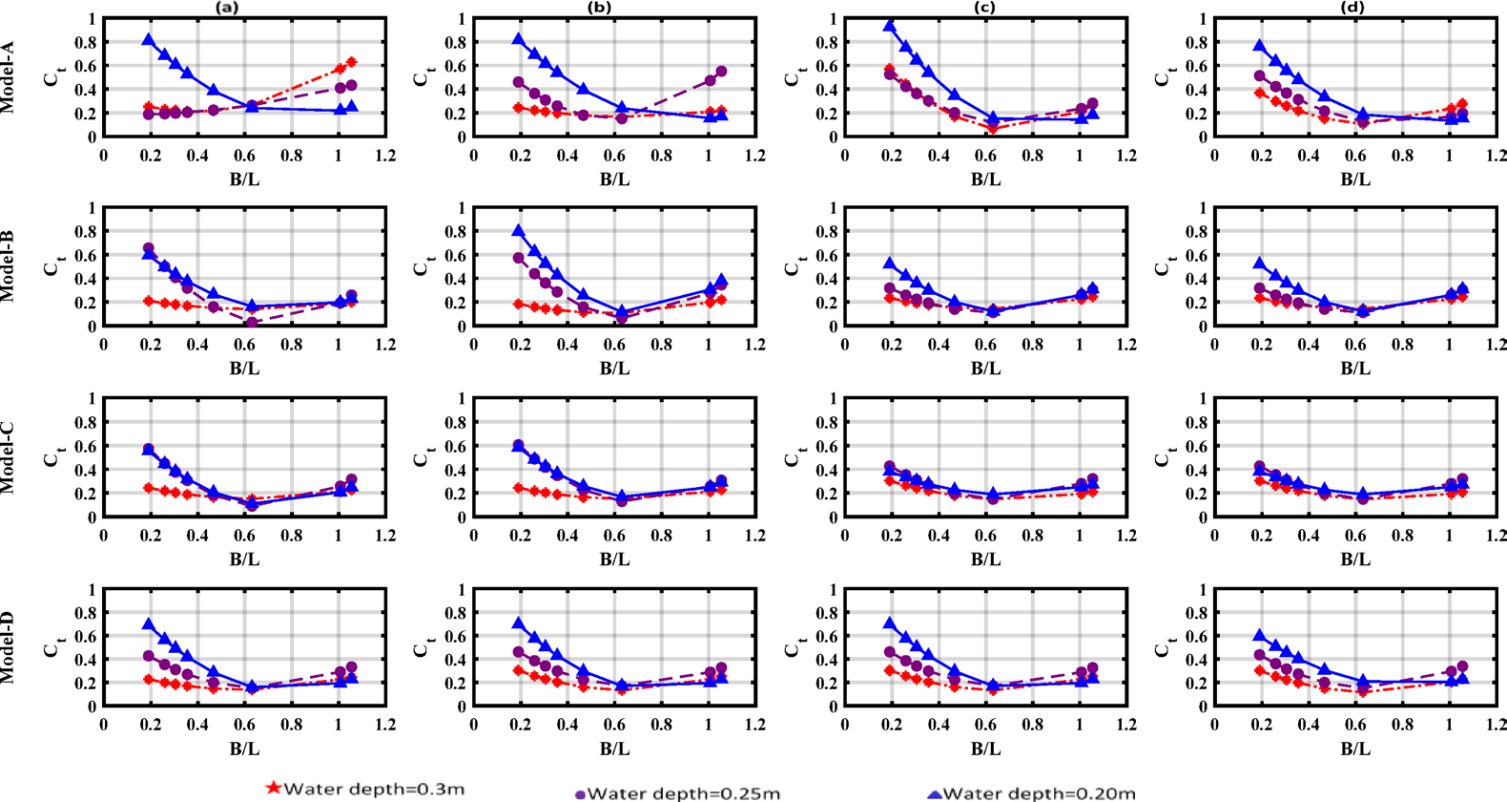
Variation of the dissipation coefficient (C_t_) with the relative width (B/L) across tested water depths; (**a**) draught = 0.04 m, (**b**) draught = 0.06 m, (**c**) draught = 0.08 m, (**d**) draught = 0.10 m.

Figure 11 illustrates the variation of the (C_t_) with the relative width (B/L) for different water depths. It is observed that (C_t_) decreases within B/L = 0.6–0.8 before increasing again.

This behavior indicates that the wave attenuation efficiency of floating breakwaters (FBs) is greater for shorter-period waves. This is expected, as shorter-period wave exhibits significantly greater interaction with floating structures. Notably, model-C and model-D demonstrated superior performance compared to the other configurations under the tested wave conditions.

Furthermore, the suppression ability of the models was observed to improve in deeper waters. Under these conditions, shorter-period waves maintain stronger surface energy concentration, allowing for more effective dissipation by pneumatic chambers, which is consistent with fixed breakwater performance^35^. Pneumatic chambers significantly reduced wave transmission coefficients across all tested wave periods. In addition to scattering effects from rear-wall configurations, transmission attenuation was achieved through: (a) reduced radiation toward the leeward side via motion suppression, and (b) enhanced energy dissipation by pneumatic chambers, which reduced both reflected and transmitted wave energy.

A (C_t_) value below 0.5 demonstrates the efficiency of the floating breakwaters. This efficiency signifies effective wave-structure interactions that decrease the intensity of the higher wave components^36^**.** This phenomenon may result from intense surface oscillations inside the pneumatic air chamber. When the wave comes against the breakwater, a sudden change in water particle motion converts to energy attenuation. Finally, steeper waves cause more turbulence, leading to sudden changes in the velocity and acceleration of water particles. This increased turbulence contributes to greater energy loss, thereby contributing to a further reduction in (C_t_).

#### Wave energy dissipation

**Fig. 12:**
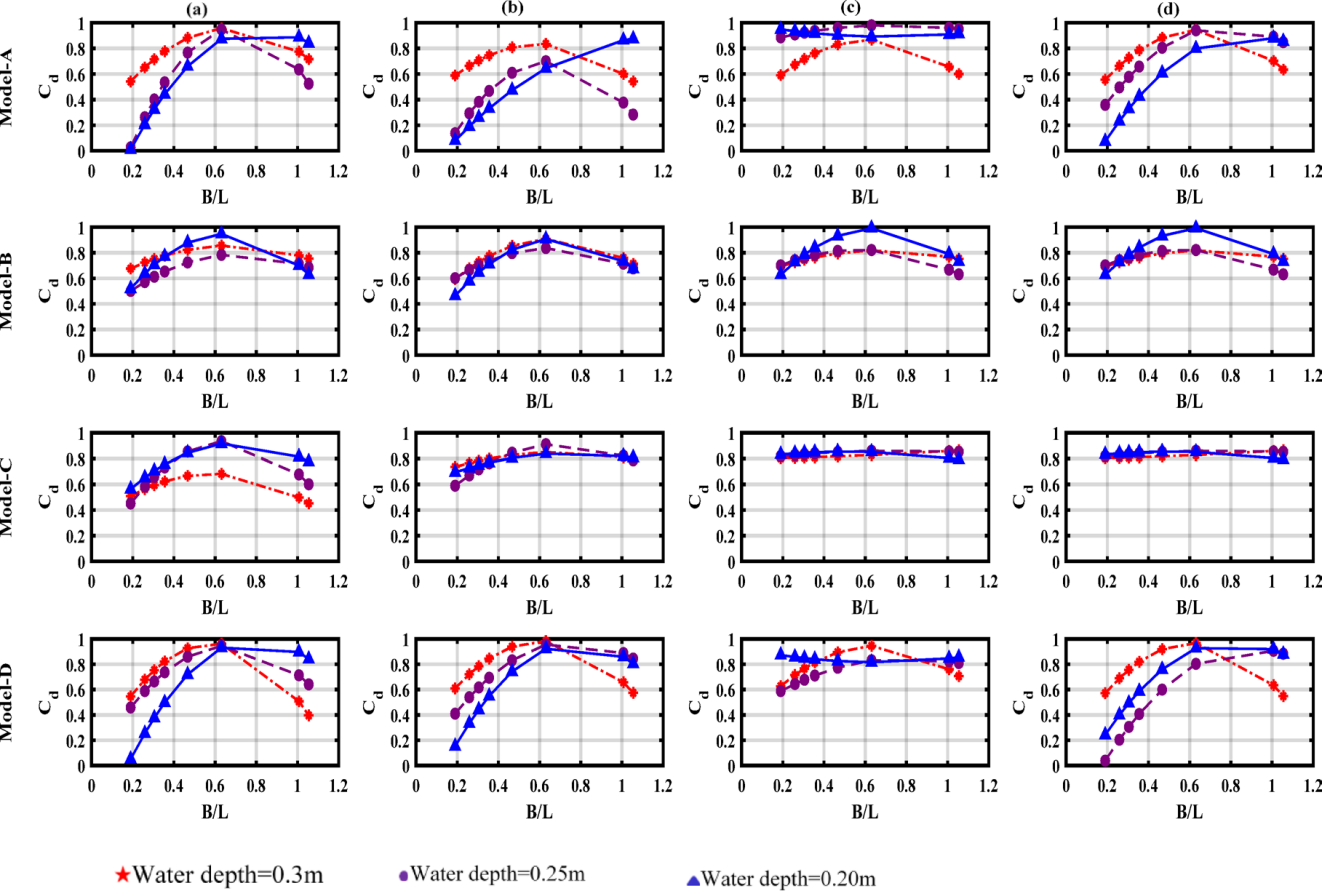
Variation of the dissipation coefficient (C_d_) with the relative width (B/L) across tested water depths; (**a**) draught = 0.04 m, (**b**) draught = 0.06 m, (**c**) draught = 0.08 m, (**d**) draught = 0.10 m.

The effect of wave height energy dissipation was initially investigated across four breakwater models. Figure 12 illustrates how the dissipation coefficient (C_d_) varies with B/L across three different water depths. For all models, (C_d_) uniformly rises with B/L, while the transmission coefficient (C_t_) decreases, indicating that the breakwaters reduce wave transmission primarily through enhanced turbulence and vortex shedding. At the same time, the reflection coefficient (C_r_) remained moderate, suggesting that energy loss is dominated by dissipation rather than reflection.

The results show that increasing the inlet depth (d_1_) enhances the efficiency of the breakwater, with optimal performance observed at the front wall draft (d_1_ = 0.1 m). In addition, deep water depth (d = 0.3 m) contributes to more efficient wave energy dissipation in all models. Among the evaluated configurations, Model-D and Model-C demonstrate the highest overall efficiency, especially at larger drafts and depths, making them the most effective for wave attenuation.

In contrast, Model-A and Model B show moderate performance, making them more suitable for shallow water and small drafts. Models C and D perform better primarily due to their enhanced design features, which enable them to dissipate wave energy more effectively.

Both models are designed for deep water and feature optimized construction designs to dissipate energy through mechanisms such as vortex buckling and friction effects. Notably, Model-D, in particular, benefits from an inclined rear wall, which increases the water column height and the natural period of the barrier, which improves its efficiency for both short and long periods. Similarly, Model-C exhibits strong performance due to its balanced design, which enables consistent efficiency across various wave conditions. In contrast, Model-A and Model-B lack the design functions required for efficient energy efficiency, such as adequate vent depth and effective air or water flow management.

This results in limited performance, especially under long-wave or deep-water conditions, making Model-C and Model-D the preferred options for optimal coastal barriers. These findings highlight the critical influence of flow dynamics and water depth on the optimal performance of wave barriers^37^.

#### Variations in air pressure within the pneumatic chambers and the wave power coefficient

**Fig. 13:**
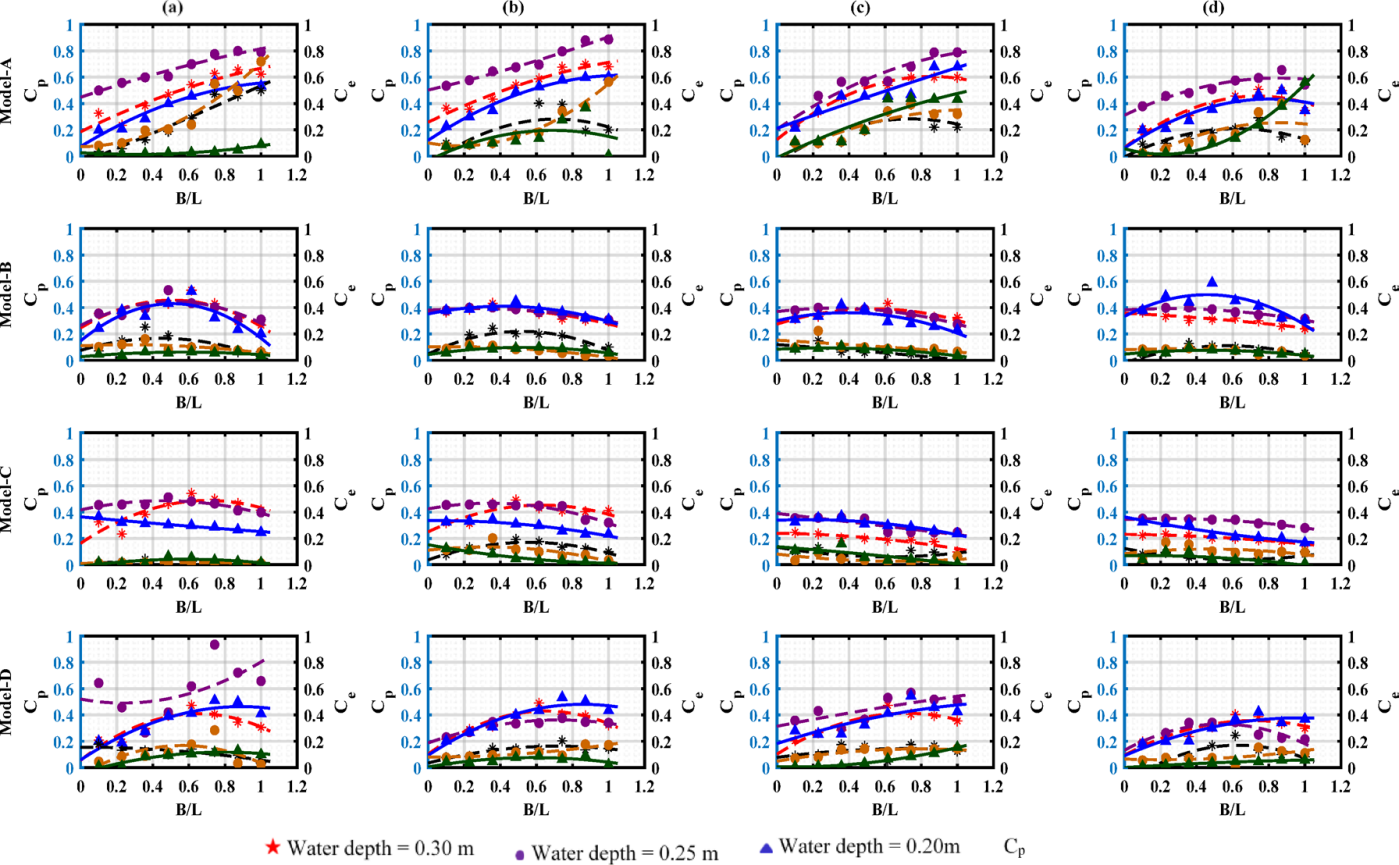
Variation of the pressure (C_p_) and the energy coefficient (C_e_) with the relative width (B/L) across tested water depths; (**a**) draught = 0.04 m, (**b**) draught = 0.06 m, (**c**) draught = 0.08 m, (**d**) draught = 0.10 m.

Variation in the dimensionless pressure fluctuation amplitude inside the chambers and the energy coefficient is plotted against B/L for four different drafts, presented in Fig. 13 . At a shallow water depth (d = 0.20 m), both the (C_e_) and (C_p_) remain relatively low across all drafts due to increased wave breaking and bottom friction, an effect most significant for smaller drafts with d_1_ = 0.04 m. As the water depth increases to d = 0.25 m, energy dissipation decreases, leading to improved performance, particularly for drafts d_1_ = 0.08 m and d_1_ = 0.10 m. At the greatest water depth d = 0.30 m, the combination of the largest draft d_1_ = 0.10 m and Model-D yields the highest of both (C_p_) and (C_e_), indicating optimal energy transfer efficiency and stable hydrodynamic performance. This analysis demonstrates the importance of deep-water depth in reducing energy losses and highlights the superior performance of large forward drafts, which minimize leakage and turbulence. Therefore, the combination of d = 0.30 m, d_1_ = 0.10 m is effective, and Model-D is the most effective configuration for increasing energy capture and efficiency.

For short waves, breakwater blockage effectively prevents wave energy transmission beneath the structure between the front and rear walls^35^. Consequently, higher water elevations produce larger strokes and compress more air within the chamber. Analysis shows that Model-D with the forward draft d1 = 0.10 m demonstrates the best performance in terms of both (C_p_) and (C_e_). For instance, at B/L = 1.0, (C_p_) and (C_e_) values reach approximately 0.85 and 0.75, respectively, indicating consistent pressure flow and efficient energy transfer. On the other hand, Model-C with d_1_ = 0.08 m achieves lower values (C_p_ = 0.8 and C_e_ = 0.7), making it less effective at larger B/L ratios, but other models perform lower; such as Model-A with d_1_ = 0.06 m reaches only (C_p_) = 0.7 and (C_e_) = 0.6. Therefore, Model-B with d_1_ = 0.10 m is considered the optimal choice for achieving the highest efficiency and dynamic performance, especially at higher B/L. The performance of the well turbine is significantly affected by factors such as the B/L ratio, structural configuration, and the pressure coefficient (C_p_), all of which affect its dynamic behavior. Initially, the Turbine energy conversion efficiency is higher as the internal chamber air pressure and pressure distribution increase. As air velocity rises, the energy coefficient (C_e_) increases gradually until it reaches a certain threshold. Beyond this point, due to elevated pressure and increased turbulence caused (C_e_), either stabilizes or declines, resulting in reduced turbine efficiency.

Table 5 presents a comparison of the hydrodynamic performance for five different WEC models, highlighting the distinct advantages of the current study’s inclined rear wall. The (C_e_) comparison showed the important energy capture advantage of the current model and the porous plate model ^9^, both achieving high C_e_ ranges (0.40–0.85) relative to the other systems, including the fixed OWC-3D^38^. Which had diminished ability to dissipate energy. The results from the current model can be directly connected to the inclined rear wall, which resulted in a more favorable level of incident wave reflection and performance (lower C_r_ values of 0.25–0.45), whereas C_r_ ≥ 0.7 for^38^;^11^. Less wave reflection therefore translates to greater incident energy into a system. Crucially, the presented breakwater operates effectively across a broader spectrum of wave conditions (B/L = 0.2–1.2), demonstrating greater operational flexibility than the fixed designs of^38^ (B/L = 0.27) and^11^ (B/L = 0.29), which are optimized for a narrow range. While porous plates^9^ also show a wide operational range (B/L = 0.19–1.055), and their wave protection capability (higher C_t_) can be less consistent. In conclusion, the data in Table 5 substantiates that the inclined wall design offers a balanced and robust solution, combining the high energy capture efficiency (C_e_) of advanced OWC systems with the excellent wave attenuation (low C_t_) and operational bandwidth necessary for effective coastal protection, outperforming traditional rigid designs and competing effectively with other advanced flexible systems.

**Table 5:** Characteristics of different laboratory types of floating breakwaters.

Reference	System description	parameters			C_r_	C_t_	C_e_
		Hi/L	d_1_/d	B/L			
Present study	Floating Breakwater	0.075–0.152	0.33	0.19–1.055	0.8–0.1	0.09–0.75	0.40–0.85
^9^	OWC with Porous Plate	0.03–0.07	0.1	0.45	0.7–0.25	0.35–0.65	0.40–0.85
^38^	Fixed OWC-3D	15.84–44.89	0.26	0.27	0.7–0.85	0.50–0.80	0.1–0.35
^11^	OWC Plate	0.03–0.07	0.1	0.29	0.80–0.45	0.40–0.80	0.20–0.50
^39^	Wave tank and OWC device	0.027–0.008	0.25	0.19–0.5	0.75–0.2	0.35–0.87	0.25–0.65

For Mediterranean-like wave conditions representative of Port Said, a significant wave height of Hi = 1.5 m and an energy period of T_e_ = 6 s was assumed. The available wave power per unit crest length is given by Eq. (13), (14), and (15^40^:

13 $${\mathrm{Hi}}^{2}\text{ x}{\text{ T}}_{\mathrm{e}}\approx {\mathrm{P}}_{\mathrm{wave}}$$

14 $${\mathrm{P}}_{\mathrm{wave}}\mathrm{x}{\text{ C}}_{\mathrm{e}}\mathrm{x}{\text{ C}}_{\mathrm{p}}\text{x B}\approx {\mathrm{P}}_{\mathrm{el}}$$

15 $${\mathrm{E}}_{\mathrm{day}}={\text{A x }24\text{ x P}}_{\mathrm{el}}$$

where Hi is wave height (m), T_e_ is energy period (s), P _wave_ is wave power (kW/m), B effective width (m), P_el_ is electrical power output, E _day_ is daily energy production, and A is availability Factor. The system delivers an electrical output of approximately 4.2 kW, which translates to nearly 86 kWh per day under an assumed operational availability of 85%. This level of energy production is sufficient to meet the demands of small-scale coastal infrastructure such as navigation aids, environmental monitoring stations, and basic desalination units. The hybrid FB–WEC system benefits from wave energy dissipation through pneumatic damping, which also reduces the breakwater displacement and construction costs. By combining coastal protection with energy generation, the system leverages cost- and space-sharing while protecting against erosion and sheltering coastal installations. The hybrid breakwater–WEC system is best suited for semi-sheltered coastal areas, such as harbor entrances, offshore islands, and Mediterranean-like coasts (e.g., Port Said).

## Conclusions

This study presents a novel design of a suspended floating breakwater with a wave energy converter (WEC). The aim was to investigate the hydrodynamic performance of various rear wall shapes with different drafts and water depths. The primary performance indicators included the wave reflection coefficient (C_r_), transmission coefficient (C_t_), dissipation coefficient (C_d_), pressure coefficient (C_p_), and energy coefficient (C_e_). The main findings can be summarized as follows:

- The geometry of the rear wall of FB-WEC significantly affects the characteristics inside the air column at different drafts. Among the four configurations, Model-D with a long slop rear wall generated the highest internal water elevation and pressure, while the other geometries produced the lowest.
- The pneumatic chambers effectively attenuated wave transmission across all tested wave period. Beyond the wave scattering induced by rear wall geometry, the decrease in wave transmission was attributed to two mechanisms: (a) the motion-generated radiate waves into the leeward side of the breakwater were lessened, and (b) additional energy dissipation and partial energy conversion into pneumatic power through the PTO system.
- The (C_r_) decreased by increasing B/L, reaching a minimum of 0.139 before rising again at smaller drafts (d_1_ = 0.04 m). Models-C and D demonstrated promising performance due to the rear wall design, which enhances energy absorption and reduces mechanical stress.
- The (C_t_) decreases as B/L increased within the range of (0.6–0.8) before rising again, indicating higher effectiveness of the floating breakwaters in attenuating shorter waves.
- Both the (C_p_) and (C_e_) increased with greater water depth, as energy losses due to bottom friction were reduced, which improves energy dissipation efficiency. The most effective configuration to maximize energy and dissipation coefficient is Model-D with a draft depth of d_1_ = 0.10. Achieving the highest (C_p_ = 0.85), and (C_e_ = 0.75), making it the optimal option for effective wave attenuation.

Model-D was identified as the optimal configuration because it achieved the highest overall conversion efficiency and stable hydrodynamic behavior compared to Models B and C. While Model-B exhibited low capture efficiency and Model-C from larger oscillations, Model-D provided a balanced performance between efficiency and stability. Notably, this performance was achieved without increasing the front width, confirming its scalability and reliability under regular wave conditions, with promising applicability to irregular sea states.

Overall, the proposed hybrid floating breakwater–WEC system demonstrates strong potential for practical deployment in coastal areas where shoreline protection and renewable energy generation are simultaneously required. In particular, semi-enclosed basins, ports, and small coastal communities in moderate-energy seas, such as the Mediterranean, are ideal locations for implementation, providing both wave attenuation and a sustainable power supply.

While the experimental results are promising, several limitations should be acknowledged. The experiments were conducted under regular wave conditions, with limited water depths, which may not fully capture the dynamic response of a realistic floating system. Moreover, the small-scale model and simplified PTO mechanism may not perfectly replicate full-scale performance.

Future work should therefore focus on large-scale experiments, testing under irregular and extreme wave conditions, and integrating a realistic mooring system and optimized PTO design to enhance the accuracy, reliability, and practical applicability of the proposed configuration.

## Data Availability

The datasets generated and/or analysed during the current study are available from the corresponding author on reasonable request.

## Abbreviations

A_0_: Swept area of the turbine [m^2^]
A: Availability Factor
a: Accuracy
B: Width of model [m]
bin: Internal width [m]
C: Blade chord [mm]
C_r_: Reflection coefficient
C_t_: Transmission coefficient
C_d_: Dissipation coefficient
C_e_: The energy coefficient
C_p_: The pressure coefficient
d: Water depth [m]
d_1_: The immersed front wall [m]
d_2_: The immersed rear wall [m]
d_0_: The diameter of pneumatic air [m]
E_day_: Daily energy production [Wh]
G: Gravitational acceleration [m/s^2^]
Ht: Transmitted wave height [m]
Hr: Reflected wave height [m]
Hi: Incident wave height [m]
He: Wave height[m]
I: Current [ Ampere]
L: The incident wavelength [m]
Le: Length of model [m]
L_p_: Prototype wavelength [m]
L_m_: Model wavelength [m]
P: Electrical power output [W]
Rh: Blade hub radius [mm]
Rt: Blade tip radius [mm]
S: Turbine blade span [mm]
Ts: Energy period[s]
t: Maximum blade thickness[mm]
U: Standard uncertainty
U_p_: Prototype velocity [m/s]
U_m_: Model velocity [m/s]
V: Velocity [m/s]
v: Voltage [Volt]
z: Number of blades
γ_w_: Specific weight of water [N/m^3^]
β: Stagger angle [degree]
ρ: Density of water, [kg/m^3^
ω: Angular speed (red/s)
BBDB: Breakwater Bent Duck Buoy
BW-WEC: Breakwater Wave Energy Converter
OWC: Oscillating Wave Column
PBBDB: Pentagonal Backward Bent Duct Buoy
PG: Pressure Gauge
PTO: Power Take Off
WEC: Wave Energy Converter
WG: Wave Gauge
